# Effect of medication timing on anticoagulation stability in users of warfarin (the INRange RCT): study protocol for a randomized controlled trial

**DOI:** 10.1186/s13063-016-1516-9

**Published:** 2016-08-04

**Authors:** Balraj S. Heran, G. Michael Allan, Lee Green, Christina Korownyk, Michael Kolber, Nicole Olivier, Mary Flesher, Scott Garrison

**Affiliations:** 1 Vancouver Coastal Health Research Institute, Vancouver, BC Canada; 2 Department of Family Medicine, University of Alberta, Edmonton, AB Canada; 3 Vancouver Coastal Health Authority, Vancouver, BC Canada

**Keywords:** Warfarin, Anticoagulant, Primary care, Vitamin K, Chronotherapy

## Abstract

**Background:**

Warfarin is an oral anticoagulant medication that disrupts the liver’s production of clotting factors. While this medication is highly effective for the prevention of thromboembolic events, it also has a narrow therapeutic range and a vulnerability to interactions with other drugs and vitamin K-containing foods. Warfarin is commonly ingested at dinnertime, the same time of day that dietary vitamin K consumption (found largely in green leafy vegetables) is most variable. While the long half-life of warfarin might make this irrelevant, the ultra short half-life of vitamin K and the possibility of a hepatic first-pass effect for warfarin make it worth evaluating whether morning ingestion of warfarin, when vitamin K levels are consistently low, leads to greater stability of its anticoagulant effect. An examination of the timing of administration on the effectiveness of warfarin has never before been conducted.

**Methods/design:**

This is a 7-month Prospective Randomized Open Blinded End-point (PROBE) study in which established evening warfarin users (primary care managed Canadian outpatients in the provinces of British Columbia and Alberta) will be randomized to either switch to morning ingestion of warfarin (the intervention) or to continue with evening use (the control). The primary outcome is the percent change in the proportion of time spent outside the therapeutic range of the international normalized ratio (INR) blood test. Secondary outcomes include change in proportion of time spent within the therapeutic INR range (TTR), percentage of patients with TTR >75 %, percentage of patients with TTR <60 %, and major warfarin-related cardiovascular events (including all-cause mortality, hospitalization for stroke, hospitalization for GI bleeding, and deep venous thrombosis/pulmonary embolism). We will also compare whether day-to-day variability in the consumption of high vitamin K-containing foods at baseline affects the baseline TTR in this cohort of evening warfarin users.

**Discussion:**

This study addresses whether the timing of warfarin ingestion influences the stability of its anticoagulant effect. Should morning ingestion prove superior, the safety and effectiveness of this medication, and hence the prevention of stroke, pulmonary embolus, and major hemorrhage, could potentially be improved with no added cost or inconvenience to the patient.

**Trial registration:**

ClinicalTrials.gov: NCT02376803. Registered on 25 February 2015.

**Electronic supplementary material:**

The online version of this article (doi:10.1186/s13063-016-1516-9) contains supplementary material, which is available to authorized users.

## Background

Vitamin K is obtained primarily from green leafy vegetables (in particular kale, spinach, chard, beet greens, broccoli, cabbage, romaine lettuce, and Brussels sprouts) [1]. It is an essential cofactor used by the liver to “activate” the clotting factors it releases into the blood. Vitamin K has a very short half-life in the body (approximately 2 ½ hours) and cycles through an active (so far as its ability to “activate” clotting factors) and inactive form in the liver [2]. Warfarin acts by preventing one of the intermediary steps necessary to convert the inactive form of vitamin K back to the active form and hence it reduces the amount of available activated clotting factor [1]. When vitamin K is first ingested, however, it is in an easily activated form upon which warfarin has little effect [2]. As a result, consumption of high vitamin K-containing foods can counteract the effect of warfarin, and highly variable consumption of these foods may cause clinically important INR variability in some individuals [3].

The effectiveness of the anticoagulant warfarin is measured by the international normalized ratio (INR) blood test, which indicates how long it takes the user’s blood to clot compared to normal [1]. Canadian warfarin users typically have their INR measured by their family physician every 1–4 weeks, and same-day changes in warfarin dosing are made in response to that day’s INR test. In order to shorten the response time for making a dosing change, patients are traditionally advised to have their INR test in the morning and to take their warfarin in the evening (so that the INR test result will be back in time to change that day’s warfarin dose if needed). However, this leads to taking warfarin at the same time of day as the most highly variable consumption of vitamin K. Conceivably, if warfarin activity is greater around the time of ingestion, when liver concentrations would be highest, taking warfarin in the morning (when the vitamin K content of typical breakfast foods is consistently low) might lead to greater stability in anticoagulant effect. The question of whether the time of ingestion of warfarin matters to INR stability has never been formally addressed.

We are conducting a pragmatic randomized controlled trial (RCT) of morning versus evening warfarin ingestion to determine whether the timing of warfarin ingestion matters to the stability of its anticoagulant effect.

### Hypotheses

Our hypotheses are as follows:

1. Morning, as compared to evening, administration of warfarin will produce a more consistent anticoagulant effect and improve the proportion of time a patient spends in the target therapeutic INR range (TTR = time in therapeutic range).
2. A less variable day-to-day dietary vitamin K consumption at dinner, whether consistently high or consistently low, will reduce variability in the anticoagulant effect of warfarin and improve the proportion of time a patient spends in the target therapeutic INR range.

### Aims

This study has the following aims:

1. To determine (by RCT) whether switching current warfarin users from evening to morning dosing will alter the proportion of time spent in the therapeutic INR range
2. To determine (by cross-sectional analysis of baseline data) whether evening warfarin users with greater variability in daily dinnertime vitamin K ingestion have a lower time in the therapeutic INR range
3. To determine by prospective subgroup analysis of RCT data whether the effect of warfarin timing on TTR (i.e., the effect of changing to morning dosing) is influenced by day-to-day variability in vitamin K consumption.

## Methods/design

### Study design

This is a Prospective Randomized Open Blinded End-point (PROBE) study [4] conforming to Consolidated Standards of Reporting Trials (CONSORT) guidelines. A CONSORT Flow Diagram is included as Additional file 1.

Ethics approval was obtained from the Health Research Ethics Board at the University of Alberta (Pro00050057) and the Clinical Research Ethics Board at the University of British Columbia (H14-03124). No modifications to the approved protocol are anticipated.

### Recruitment

Participating community family physicians in the Canadian provinces of British Columbia and Alberta will send a letter of invitation to all warfarin-using patients under their care with the exception of those whom they view as palliative or deemed incapable of informed consent. This letter: (1) describes the project, (2) lets patients know their physician’s office is participating, and (3) provides a central contact number that reaches our study coordinator for more information if they are interested. The study coordinator dialogs with interested patients who call in, screens them for eligibility, and obtains written informed consent (via online REDCap survey or mail-in consent form) from all patients willing to be randomized. For consenting participants who meet the screening criteria for eligibility, the family physician will be asked to provide investigators with the participant’s target INR range and their last 6 months of INR results, test dates, and warfarin dosing.

### Data collection and management

Study data will be collected, managed, and securely maintained using REDCap electronic data capture tools hosted by the Women and Children's Health Research Institute at the University of Alberta [5]. Non-electronic personal information of potential and enrolled patients (consisting of INR results and INR flow sheets faxed to us by family physicians) will be kept in a locked fireproof cabinet accessible only to the study investigators and staff. All study data (including INR results) will be archived in REDCap and maintained for a minimum of 5 years.

### Inclusion and exclusion criteria

Study inclusion criteria include: (1) dinner or evening use of warfarin, (2) ≥3 months of continuous warfarin use, (3) expectation of long-term warfarin use, (4) baseline INR data (last 6 months if available, minimum 3 months) with at least four evaluable INR results no more than 8 weeks apart provided to the study team by patient’s family physician, and (5) community dwelling. Exclusion criteria include patients under palliative care or those who are unable to provide informed consent in the opinion of the treating family physician.

### Randomization and allocation

Upon receiving eligible baseline INR data, consented eligible participants are contacted by a study coordinator via telephone to obtain baseline information presumed to be predictive of TTR. This includes age ≥80 years, hospitalization in the last 6 months, temporary planned discontinuation of warfarin in the last 6 months (e.g., for elective surgery), number of daily prescription medications, <6 months of warfarin use, the self-reported average number of days per week in which high vitamin K-containing foods are consumed, and how variable the participant feels this estimated level of vitamin K consumption is (on a 4-point scale). This study coordinator (who has no clinical patient interactions) then randomizes the participant, using the REDCap^TM^ randomization module to ensure allocation concealment, to intervention or control [5]. Randomization will employ variable blocks of 2 or 4 and will be stratified by the proportion of baseline INR readings within the therapeutic range (<50 %, 50–80 %, >80 %).

### Intervention and blinding

Active arm subjects will switch their warfarin use to morning. The control arm will continue with their current pattern of evening use. There are no run-in or washout periods in this study. Participants randomized to morning warfarin ingestion will be asked to make the change 5 days prior to their next scheduled INR test (so that any deviation in the INR can be detected early). Although patients and their physicians will be aware of treatment assignment, our study evaluators will be blinded to allocation.

### On-study participant interaction

Participants have the option to interact with study staff either online (via online consent and online follow-up surveys using REDCap) or in person (via mailing in consent and having follow-up interviews over the phone where study staff record their responses in REDCap). Following the randomization telephone interview, such online or in-person follow-up interviews then occur at 1 week, 1 month, and 7 months (relative to the expected date of first morning ingestion of warfarin in those allocated to morning use, or relative to the date of randomization in those allocated to control). During these interviews participants self-report their adherence to the allocated timing of warfarin ingestion, as well as any illnesses and potentially warfarin-related adverse events (bleeding and thromboembolic events). Patients are encouraged to adhere to the allocated intervention during telephone follow-up, and suggestions to facilitate adherence at the time of allocation include: (1) helping to set a routine by placing their warfarin near objects that they interact with at the relevant time of day (e.g., other medications, toothbrush, false teeth, coffee pot/kettle/toaster), (2) using a dosette box with time of day divisions, (3) setting an alarm, (4) asking their spouse to remind them, and (5) offering to have study staff remind them the day before any switch to morning warfarin is supposed to occur. Decisions on discontinuing warfarin or returning to evening dosing in response to perceived harm, or upon patient request, will be made entirely by the treating physician at their discretion. The same is true for any decisions related to concomitant care. Participants are free to withdraw from the study at any time without penalty and can decide to have their data excluded from, or continue to be included in, the analysis.

### Outcomes

Seven months after randomization of each patient, we will ask their family physician to provide us all INR results for that period in order to determine the following outcomes during the last 6 months of therapy.

#### Primary outcome

The primary outcome is percentage change in time spent *outside* the family physician’s target INR range for that patient. This primary outcome has been chosen because we believe this is a measure more likely to be shared by patients across a wide range of TTR. It is also the time spent outside of range that contributes more directly to risk of thrombosis and hemorrhage; hence, the change in this measure is more clinically meaningful than the change in TTR itself.

#### Secondary outcomes

The secondary outcomes are:

1. Change in TTR
2. Percentage of patients with TTR >75 %
3. Percentage of patients with TTR <60 %
4. For those patients with at least one INR value above the therapeutic range, the maximum INR value observed
5. For those patients with at least one INR value below the therapeutic range, the minimum INR value observed
6. Percentage of time spent above the therapeutic range
7. Percentage of time spent below the therapeutic range
8. Major warfarin-related cardiovascular events (including all-cause mortality, hospitalization for stroke, hospitalization for GI bleeding, and deep venous thrombosis/pulmonary embolism)

The percentage of patients with TTR >75 % provides the percentage of patients considered to have excellent control, while the percentage of patients <60 % provides the percentage of patients for whom other anticoagulation strategies may be indicated [6, 7]. Maximum and minimum INR values provide a sense of how far out of range patients go, while major warfarin-related cardiovascular events aggregate outcomes that might stem from both inadequate and excessive anticoagulation.

#### Safety outcomes

The safety outcomes, as initially flagged from patient interviews and confirmed with family physicians, are:

1. Major thromboembolic events (including non-hemorrhagic stroke, deep vein thrombosis, pulmonary embolus, and acute arterial occlusion)
2. Major bleeding events (includes all bleeding events requiring hospitalization or transfusion such as hemorrhagic stroke and GI bleeding).

As this study is small, relatively low risk, and of short duration, there will be no interim safety analysis and hence no formal data safety monitoring board.

The occurrence of major adverse events will be reported to the principal investigator, who will determine the appropriate action should a signal of harm in the intervention group arise.

### Sample size

We wish to be able to demonstrate a 20 % reduction in time out of therapeutic range and will conservatively estimate (since there are no prior studies exploring this outcome to guide us) that the standard deviation of this measure is twice the mean effect (i.e., SD = 40 %). For a *t* test with 1:1 allocation to the control and experimental groups, power = 0.9, alpha = 0.05, minimum difference = 20 %, and SD = 40 %, the required sample size per group is 85 (i.e., 170 subjects in total). Providing for potential dropouts, we will increase our target enrollment to 200 subjects.

### Statistical analysis

#### Calculating TTR

The therapeutic INR range for each patient will vary and be determined by the treating physician. Typically the therapeutic range is 1 unit wide (often 2–3 or 2.5–3.5), but some physicians will choose narrower or wider ranges (e.g., 3.0–3.5 or 2–3.5). We will standardize the width of all target ranges by determining the midpoint of each patient’s individual target range and use upper and lower limits that are 0.5 unit above and below this midpoint. For example, if a physician is targeting a narrower than normal 3.0–3.5 range, we will use a midpoint of 3.25 and assume a (standardized width) target therapeutic range of 2.75–3.75. The proportion of time both in and out of therapeutic range will be determined using the linear interpolation method of Rosendaal, which (conceptually) draws a line between sequential INR values no more than 8 weeks apart and assigns a projected INR value to every day in that interval [8].

#### RCT

All analyses will be by intention to treat. The primary analysis of percentage change in time outside of therapeutic range will be by Student’s *t* test if the data appear to be normally distributed or by Mann-Whitney U test if they are not. As percentage change in time outside of therapeutic range can only be calculated for patients for whom the time outside normal range is not zero, this analysis will exclude anyone who was in range 100 % of the time at baseline. The secondary analyses will be by Student’s *t* test or Mann-Whitney U test (for percentage change in TTR, maximum and minimum INR values, and percentage of time spent above and below target range) and by Student’s *t* test or Fisher’s exact test (for both percentages of patients with TTR >75 % and <60 %, and major warfarin-related cardiovascular events). A subgroup analysis of the influence on the intervention of the numbers of days per week that high vitamin K foods are ingested will be carried out looking at an ANOVA analysis of percent change in time outside of therapeutic range according to three possible categories for the number of days per week of consumption of high vitamin K-containing foods (these being less than 2, 2 to 5, and greater than 5 days per week). In the same analysis we will also examine the effect on the intervention of the patient’s global assessment of how variable their consumption of high vitamin K containing foods is. To do this, we will convert the 4-point scale of possible responses into a dichotomous variable that combines the two options indicating the most variable diet and the two options indicating the least variable diet.

#### Baseline cross-sectional analysis

The effect on baseline TTR of the number of days per week that high vitamin K-containing foods are consumed will be analyzed with multiple linear regression using baseline covariates which include: gender, age ≥80 years, hospitalization in the last 6 months, temporary planned discontinuation of warfarin in the last 6 months, number of daily prescription medications, <6 months of warfarin use, each of the three possible categories for number of days per week consuming high vitamin K-containing foods, and the patient’s dichotomous global assessment of how variable their pattern of vitamin K consumption is.

## Discussion

There are no studies or systematic reviews evaluating the optimal timing of warfarin dosing. The assumption that evening use will lead to more stable INR management (by more prompt dose adjustment) has never been tested. Given that vitamin K interferes with the effectiveness of warfarin, it is conceivable that matching the time of greatest warfarin activity to the period of most consistent vitamin K availability (i.e., taking warfarin at breakfast) might lead to greater stability in warfarin's anticoagulant effect.

Warfarin is widely prescribed (often to older adults with multiple comorbidities), and patients receiving this medication are most commonly managed by their family physician. Conducting this pragmatic trial in “real-world” community primary care practices serves to maximize the generalizability of our findings.

This study has the potential to benefit both our study participants and the population of warfarin users worldwide. This potential benefit must be weighed against the potential risk to participants (during the 7 months of trial participation) from an anticipated extra 8 to 12 hours delay between learning the results of an INR test and adjusting a dose accordingly. Because the usual interval between tests is 1 to 4 weeks, and given the short duration of this trial, we believe this is not an unreasonable risk when weighed against the potential benefit of improved prevention of stroke and other thromboembolic events over the longer term.

### Dissemination of findings

An interactive executive summary webinar will be made available to all trial participants consenting to be contacted with our study findings, and trial results will be posted on the Pragmatic Trials Collaborative website for the public to peruse. All participating clinics, physicians, and consenting participants will additionally be informed of the results via email or letter mail. If the trial findings are clinically impactful, they will be more broadly disseminated via the Best Science Medicine Podcast and Tools for Practice evidence summaries, which are emailed directly to subscribing family physicians (both of these knowledge translation vehicles are co-produced by members of our investigative team). The Pragmatic Trials Collaborative will further disseminate any meaningful findings to its physician members and to its supporting partner organizations. Regardless of the impact of the findings, a manuscript will be prepared and submitted to a peer-reviewed journal. Upon publication of the last planned manuscript stemming from this work, anonymized patient level data will be made available online for use by other researchers.

### Trial status

Recruitment started in February 2015. Patients are still being recruited at the time of submission.

## Abbreviations

INR, international normalized ratio; TTR, proportion of time in therapeutic range

## Additional file

Additional file 1: INRange CONSORT flow diagram. (DOCX 191 kb)
