# Supplementary material for: Effect of medication timing on anticoagulation stability in users of warfarin (the INRange RCT): study protocol for a randomized controlled trial
Source: Trials. 2016 Aug 4;17:391. doi: 10.1186/s13063-016-1516-9 (PMC4973068; doi:10.1186/s13063-016-1516-9)
Supplement: Additional file 1: — INRange CONSORT flow diagram. (DOCX 191 kb) [file 13063_2016_1516_MOESM1_ESM.docx]

**INRange RCT – CONSORT Flow Diagram**

**Adverse Event reported** -

Notify Principal Investigator and take necessary action. May or may not involve close out

**TASK LEGEND:**

**Study staff**

**Site (family physician) staff**

**Principal investigator**

**Inside study step**

**Blinded study step**

Review INR data for completeness and eligibility ,

calculate % INR values in target range for randomization stratification

**Mail-in consent:** consent form mailed back

OR

**Online Consent:** via REDCap survey

Recruitment site mails information

packages to eligible patients

Fails screening

(1-2 mins)

Patient initiated call, study info discussed, screening & informed consent (5-10 min)

Fails Consent – contact to confirm & go to **Close out**

(1-2 mins)

Review consent & request INR data from clinic / physician

Insufficient INR data – call participant to **Close out**

(1-2 min)

Declines Randomization **Close out** (1-2 min)

Telephone call to perform baseline & randomization interview (20min)

**SCREENING & CONSENT**

**RECRUITMENT**

Receive INR data **- Successful study**

**close out completed**

Contact clinic / physician for last 7 months INR data

Follow up interviews:

1 week (1-3 min)

1 month (1-3 min)

7 month (15 min)

+/- illness/AE if reported

**FOLLOWUP**

**CLOSE OUT**

Declines participation during interview - **Close out**

(1-2 min)

**ALLOCATION**
